# Board of Naval Surgeons

**Published:** 1843-03-18

**Authors:** 


A Board of Naval Surgeons assembled at the Naval Hospital (Asylum) Philadelphia, composed of Thomas Dillard, (President,) W. S. W. Ruschenberger, Samuel Barrington, Wm. Maxwell Wood and Daniel Egbert, (Members,) for the examination of Assistant Surgeons for promotion, adjourned on Friday, the 3d of March.

The following is a list of the successful candidates, with the date of their respective commissions, and the subjects of their theses before the Board, arranged in the order of merit.

| | *Date of Com.* | *Subject of Thesis.* |
|---|---|---|
| James C. Palmer, | Mar. 26, 1834, | Diseases of the Liver. |
| John L. Fox, | Feb. 9, 1837, | Medical Topography, and diseases of the Fejee Islands. |
| Charles F. Guillou, | Do. | Gonorrhœa. |
| John T. Mason, | Sept. 6, 1837, | Bloodletting, its uses and abuses in the practice of medicine. |
| Charles D. Maxwell, | Do. | Rheumatism. |
| Edward J. Rutter, | Do. | Effects upon the functions of the brain produced by external injuries. |
| John J. Abernethy, | Do. | Fractures of the Femur. |
| James Malcolm Smith, | Do. | Dislocations of the Femur. |
| Charles Wm. Tait, | Do. | The best means of preserving the health of Seamen. |

It will be observed that all the above named candidates have been six years, and one of them nearly nine years in the Navy.

The subject of the thesis is assigned by the Board; and it is immediately written by the candidate in an adjoining apartment. A single sheet of foolscap paper is given to each, so that the opportunity of copying is cut off; nothing but previous knowledge, and a well trained habit of thought, will enable the candidate to produce a reasonably respectable treatise, written under these difficulties and disadvantages.

